# Education General and Medical

**Published:** 1906-09-08

**Authors:** 


The Hospital

A Journal of

The Medical Sciences and Hospital Administration.

Vol. XL.—No. 1,042. SATURDAY, SEPTEMBER 8, 1906.

### Education—General and Medical.

The aspect of the recent meeting of the British Association which, apart from the numerous papers dealing with questions of physical and biological science, will appeal most strongly to members of the medical profession is to be found in all probability in the various discussions on the problems connected with educational efficiency. The principles and methods defined in these debates concern, indeed, every citizen, and more particularly those who have assument parental responsibilities. But they must excite the especial interest of the members of a profession to which the general mass of the public naturally turns for guidance in reference to the healthy and vigorous development of the physical and mental qualities of the rising generation. Still further, the educational methods at present in force as a scheme of training for entrance to the medical profession are not by any means universally regarded as satisfactory. And, criticism apart, it is obviously possible that discussions on education in general may readily reveal facts and doctrines which will prove helpful to remove the difficulties that attend the problem of medical education in particular.

To suggest that nothing can be more vital to educational success and efficiency than the careful selection and adequate training of teachers would seem, at first sight, to be an obvious and weary platitude. Yet it is certain that the public in general have but a faint appreciation of this very elementary proposition. It may be too much to say, as was said in one of the discussions at York, that education makes the man; but it undoubtedly has a large share in the making of men. If this is so there can be no class or order of more importance to the welfare of the body politic than that of the teacher. How far this is from being recognised is illustrated by the arrangements which leave members of the teaching profession as the least considered and worst paid of any class of public servants. Until public opinion becomes enlightened on this point and is willing to render to the teacher the honour and recognition that are his due, it is hopeless to expect that the best minds will be attracted by the laborious but fruitful opportunities of the teaching profession. But if this criticism applies to education in general, it certainly also applies to the conditions of our medical schools. It is notorious that in these institutions teachers are, at least very often, appointed by the most haphazard methods. For some reason or other, often no doubt a very good reason, a junior graduate gains an appointment as a member of a staff of a hospital associated with a medical school. His capacity as a teacher is probably quite unknown, and, in any case, receives little consideration. Yet he is straightway called upon to lecture on some subject which happens to present a vacancy and to which he has probably never given the slightest consideration since the day he passed his final examination. The process is completed by the prompt election of the new teacher to one of the examining boards, and this the more certainly if he has colleagues who exercise influence with the examining corporations. Thus, as is well-known, our medical schools present the names of men who may be, and often are, efficient, and even distinguished physicians and surgeons, but who have never enjoyed the slightest training as teachers, and who in this capacity are melancholy and notorious failures. Bad as this is in itself the worst feature is that it hardly excites comment or anxiety. It is treated as a necessary part of an imperfect world, to be accepted with a smile and a shrug of the shoulders. And for all this there is neither help nor redress so long as the medical profession shares the attitude and conviction of the general public that teaching ability, efficiency, and industry merit no particular recognition and are not worthy of special appreciation and reward.

Another question raised in the recent discussions was that of the value and function of the inspector of schools. It is not perhaps difficult to understand why proposals from various quarters should suggest that these officials should be abolished, and in so far as they interpret their duties in a narrow, dictatorial, and censorious spirit, they probably do more harm than good. But, as pointed out in an admirable paper by Mr. W. Marghowe Heller, the practice of inspection, if pursued with an instructed and lofty motive, is competent to bring to teachers what they need more than criticism and censure—namely, suggestion, help, and encouragement. Only those who have wide and adequate opportunities for observation and comparison are in a position to supply those hints for development or improvement which are so welcome to all true workers, and from this point of view the work of a competent and sympathetic inspector may be most valuable and helpful. Without it, indeed, there is a distinct danger of the development of narrow habits and crude routine. Mutual conference between teachers, and in reference to the aims and methods of their work, is another influence recognised as necessary to the diffusion of a high standard of efficiency. In view of all this it may well be asked whether medical teaching might not with advantage be subjected to similar agencies. Inspection of examinations is a recognised practice. Why not also of medical teaching? The examiner does not feel his dignity compromised by such a measure; nor need the conscientious and earnest teacher. Indeed, whatever exalts the position and responsibility of the teacher and emphasises the demand for thoroughness and efficiency would, we are convinced, be in the interests of medical education, and individual comment and general conference may certainly help to contribute to these ends.

